# Neurotoxicity in complex environmental mixtures—a case-study at River Danube in Novi Sad (Serbia) using zebrafish embryos

**DOI:** 10.1007/s11356-023-29186-1

**Published:** 2023-08-11

**Authors:** Riccardo Massei, Werner Brack, Sven Seidensticker, Henner Hollert, Melis Muz, Tobias Schulze, Martin Krauss, Eberhard Küster

**Affiliations:** 1 Department of Bioanalytical Ecotoxicology, UFZ—Helmholtz Centre for Environmental Research, Leipzig, Germany; 2 Department of Effect-Directed Analysis, UFZ—Helmholtz Centre for Environmental Research, Leipzig, Germany; 3 Department of Evolutionary Ecology and Environmental Toxicology, Faculty of Biological Sciences, Goethe University Frankfurt, Frankfurt, Germany; 4 Department of Monitoring and Exploration Technologies, UFZ—Helmholtz Centre for Environmental Research , Leipzig, Germany; 5 LUFA Nord-West, Institut für Lebensmittelqualität, Oldenburg, Germany

**Keywords:** Risk assessment, Wastewater, Enzyme biomarker, Effect-directed analysis (EDA), Pollution

## Abstract

Acetylcholinesterase (AChE) inhibitors are an important class of neuroactive chemicals that are often detected in aquatic and terrestrial environments. The correct functionality of the AChE enzyme is linked to many important physiological processes such as locomotion and respiration. Consequently, it is necessary to develop new analytical strategies to identify harmful AChE inhibitors in the environment. It has been shown that mixture effects and oxidative stress may jeopardize the application of in vivo assays for the identification of AChE inhibitors in the environment. To confirm that in vivo AChE assays can be successfully applied when dealing with complex mixtures, an extract from river water impacted by non-treated wastewater was bio-tested using the acute toxicity fish embryo test (FET) and AChE inhibition assay with zebrafish. The zebrafish FET showed high sensitivity for the extract (LC10 = relative extraction factor 2.8) and we observed a significant inhibition of the AChE (40%, *p* < 0.01) after 4-day exposure. Furthermore, the extract was chromatographically fractionated into a total of 26 fractions to dilute the mixture effect and separate compounds according to their physico-chemical properties. As expected, non-specific acute effects (i.e., mortality) disappeared or evenly spread among the fractions, while AChE inhibition was still detected in five fractions. Chemical analysis did not detect any known AChE inhibitors in these active fractions. These results confirm that the AChE assay with *Danio rerio* can be applied for the detection of neuroactive effects induced in complex environmental samples, but also, they highlight the need to increase analytical and identification techniques for the detection of neurotoxic substances.

**Supplementary Information:**

The online version contains supplementary material available at 10.1007/s11356-023-29186-1.

## Introduction

It has been recently estimated that up to 30% of all commercial organic chemicals detected as micropollutants can target the central and peripheral nervous system (Legradi et al. 2018). These pollutants, here defined as neuroactive, are known to negatively affect aquatic organisms by causing developmental neurotoxicity, disturbances in electric signal transduction, inhibition of chemical signal transduction, and behavioral impairment at environmental relevant concentrations (Casida 2009; Casida and Durkin 2015; Legradi et al. 2018; Leuthold et al. 2018). In particular, acetylcholinesterase (AChE) inhibitors represent a broad group of neuroactive chemicals which can enter the environment through several anthropogenic sources such as agriculture, private gardening, veterinary, and medical practices. These pollutants are capable of inhibiting AChEs, cholinesterase enzymes present in neuromuscular synapsis that terminate neurotransmission via hydrolysis of acetylcholine. It has been shown that a correct functionality of the AChE is important for many key functions such as locomotion, predator evasion, prey location, orientation toward food and feeding, spatial distribution pattern, and social interactions (Dutta and Arends 2003). Prolonged inhibition of AChE can cause cholinergic crises, over-stimulation of the post-synaptic neurons leading to paralysis, respiratory failure, and, in extreme cases, death of the organism (Fu et al. 2018). Considering their potential risk toward aquatic organisms, it is necessary to develop analytical strategies to identify AChE inhibitors in the environment.

In this context, routine chemical monitoring is a valid strategy to identify specific AChE inhibitors in different environmental matrices. However, analytical approaches based on measuring a limited number of target chemicals may overlook undetected toxicologically relevant compounds and associated transformation products and, most important, underestimate mixture effects (Altenburger et al. 2019; Brack et al. 2019). Moreover, compounds with highly specific modes of action (MoA) can exhibit adverse effects at concentrations far below instrumental quantification limit. Bioanalytical tools can overcome these limitations. In particular, the fish embryo test (FET) with the zebrafish (*Danio rerio*) is a powerful in vivo tool capable of identifying the effects of toxicants on the whole organism (OECD 2013; Nagel 2002). Furthermore, the AChE inhibition assay with zebrafish embryos, a sub-lethal assay widely applied for the detection of AChE inhibitors in environmental samples (Kais et al. 2015; Massei et al. 2019; Stengel et al. 2018; Velki et al. 2017), can be applied for the detection of neurotoxicity. However, it has been shown that AChE activity can be affected by different non-neuroactive chemicals. Common wastewater pollutants such as surfactants, metals, and even PAHs can affect the AChE activity in vivo (Schmidel et al. 2014; Guilhermino et al. 2000; de Lima et al. 2013; Kang and Fang 1997). Furthermore, unspecific effects (i.e., neurodevelopmental toxicity) may jeopardize the application of the assay for the identification of AChE inhibitors in complex environmental mixtures.

This study aimed to understand the application of the AChE assay with zebrafish embryos to detect AChE inhibitors in complex environmental mixtures. To this end, we selected an environmental extract which was already chemically characterized in previous studies (Hashmi et al. 2018; König et al. 2017). This extract was selected since chemical analyses confirmed the presence of several organic micropollutants (OMPs) including AChE inhibitors. As a first step, the extract was tested for acute toxicity using the FET along with the AChE inhibition assay with zebrafish. In a second step, the extract was chromatographically fractionated to show the applicability of the AChE assay with zebrafish to identify AChE inhibitors in complex mixtures. Chromatographic fractionation is a procedure leading to the separation of chemicals according to their physico-chemical properties. Since unspecific effects such as lethality occur due to exposure to very broad range of compounds, fractionation typically results in the distribution of toxicity over many different fractions (Di Paolo et al. 2015). This phenomenon has been observed previously for other bioassays detecting mainly unspecific effects (Reineke et al. 2002). In contrast, specific effects such as AChE inhibition should be detectable by the AChE assay in a limited number of individually fractions. This principle is used in the current paper to demonstrate the potential of the AChE assay to detect this specific effect even in complex mixtures. The AChE assay was supported by chemical analysis to target causative AChE inhibitors in active fractions (i.e., fractions which showed effects in the AChE assay) and exclude as much as possible chemicals leading to unspecific effect.

## Material and methods

### Sampling and sample fractionation

**Fig. 1:**
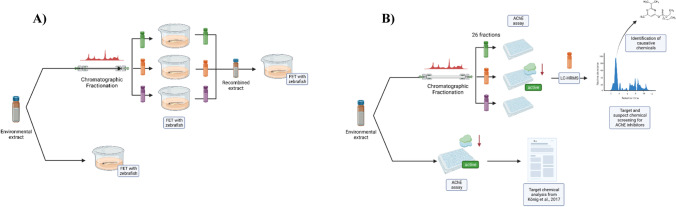
Overview on the experimental design for **A** the fish embryo test (FET) with zebrafish and **B** the acetylcholinesterase (AChE) assay with zebrafish. Figures included the fractionation and chemical analysis steps. Both figures were created with BioRender.com

River water was sampled in November 2014 in a plume of untreated wastewater discharged (45° 15′ 12.5″ N, 19° 51′ 21.0″ E) into River Danube at the city of Novi Sad (Republic of Serbia). This site is characterized by high concentrations of several OMPs and AChE inhibitors (Čelić et al. 2020; Hashimi et al., 2018; König et al. 2017). Shortly, 850 L of water was extracted on-site for multiple studies and purposes by large-volume solid phase extraction (Schulze et al. 2017; Välitalo et al. 2017) on a Chromabond® HRX sorbent, a hydrophobic polystyrene-divinylbenzene copolymer (Macherey–Nagel, Düren, Germany) of which an aliquot was used for the present study. The concentrated extract (1000-fold) was fractionated (26 fractions altogether) according to Hashmi et al. (2018) using reversed-phase high-performance liquid chromatography (RP-HPLC) with few modifications. The extracts and fractions were stored at − 20 °C in methanol and dark condition until testing to avoid chemical degradation. Further details about sampling and sample preparation are described by Hashmi et al. (2018) and König et al. (2017). Further information regarding fractionation and fraction preparation can be found in the Supplement Data (Annex 1). An overview on our experimental approach is given in Fig. 1. In order to avoid chemical degradation due to storage, biological analysis (FET and AChE assay) was performed within 1 year after sample collection.

### Target and suspect chemical analyses (LC-HRMS)

Target chemical analysis of the sample was performed by König et al. (2017). The targeted screening included 276 emerging contaminants from different classes (i.e., pesticides, personal care products, and industrial chemicals) known to occur in surface and wastewater. Among the 125 compounds detected, chemical screening highlighted the presence of 8 AChE inhibitors at concentrations between 3 (carbaryl) and 4000 ng/L (caffeine) (Table 1). In the present study, active fractions (defined as fractions showing significant statistical differences in effects with respect to control (*p* < 0.05) in the AChE inhibition assay) were subjected to chemical target screening for the AChE inhibitors detected by König et al. (2017) by using liquid chromatography–high-resolution mass spectrometry (LC-HRMS). To increase the number of screened chemicals, a screening of an additional 22 organophosphate and carbamate insecticides known to be used in European agriculture was conducted using LC-HRMS. Candidate chemicals for suspect screening were selected based on an information retrieved from different sources (i.e., literature research, online databases, expert judgement) and appear in the Supporting Information (Table S1). Detailed information regarding target and suspect chemical analysis can be found in the Supplement Data (Annex 1).

**Table 1:** List of acetylcholinesterase inhibitors detected in the untreated waste water of Novi Sad by König et al. (2017)

Compound	Concentration (ng/L)	Usage	Inhibition mechanism
Caffeine	4000	Food additive	Non-competitive inhibitor
Carbaryl	3	Pesticide	Reversible inhibitor
Carbendazim	12	Pesticide/biocide	Reversible inhibitor
Icaridin	3.4	Biocide	Reversible inhibitor
Diazinon	6.2	Pesticide	Irreversible inhibitor
Ethyl-azinphos	3.4*	Pesticide	Irreversible inhibitor
TBOEP	44	Flame retardant	Irreversible inhibitor
TCEP	38	Flame retardant	Irreversible inhibitor

^*^Compound was detected below the method detection limit (MDL)

*TBOEP*, tris(2-butoxyethyl) phosphate; *TCEP*, tris(2-chloroethyl) phosphate

### Zebrafish fish embryo test (FET)

Zebrafish of the strain UFZ-OBI (WIK wild type) were cultured and used according to German and European animal protection standards and approved by the Government of Saxony, Landesdirektion Leipzig, Germany (Aktenzeichen 75–9185.64). Fish were kept in 14-L aquaria with 25–30 fish each with a sex ratio of female to male of 1:2. The light–dark rhythm was 14:10 h and the water temperature was 26 ± 1 °C. Water parameters were measured monthly (pH: 7–8; water hardness: 2–3 mmol/L, conductivity: 540–560 µS/cm, nitrate: ≤ 2.5 mg/L, nitrite: ≤ 0.025 mg/L, ammonia: ≤ 0.6 mg/L, oxygen saturation: 87–91%) and fish were fed twice daily with fresh *Artemia salina*. Within 30 min of spawning, eggs were collected using a grid covered dish and successively cleaned with aerated ISO standard dilution water (ISO water) as specified in ISO 7346–3. Developmental stages were identified according to Kimmel et al. (1995). The zebrafish acute FET was started shortly after fertilization of the eggs (0–2 h post fertilization, hpf). Fertilized eggs were separated from non-fertilized eggs after 1 h (four to eight cell stages). Non-fertilized eggs and eggs with obvious irregularities or injuries were excluded. The viable fertilized eggs were washed with ISO water and transferred to 25-mL glass Petri dishes (7 cm in diameter, VWR, Darmstadt, Germany)—glass Petri dishes were used to decrease the possibility of sorption of lipophilic substances to the test chamber. In total, we exposed 10 embryos with the ratio of 1 embryo/mL of exposure media (final volume: 10 mL). Exposure was started not later than 2 hpf according to OECD 236 guidelines (OECD 2013) and the overall test duration was 96 h. Zebrafish less than 120 hpf are still feeding on their yolk and, hence, are not considered animals. Thus, no animal test authorization was required according to European legislation (Strähle et al. 2012).

Every 24 h, the embryos were checked for apical endpoints indicative of mortality (i.e., coagulation of fertilized eggs, lack of somite formation, lack of detachment of the tail-bud from the yolk sac, and lack of heartbeat) and sub-lethal endpoints described in the OECD 236. Dead embryos were removed daily to avoid fungal growth. The glass Petri dishes were stored in an incubator at 27 °C ± 1 with a light–dark cycle of 14:10 h and were placed on a shaker at 45 rpm to increase time to equilibrium between organisms and exposure media (Schreiber et al. 2009). Test concentrations were calculated as relative enrichment factors (REFs) which describe how much the water sample was enriched prior to the FET and have the net units of Volume_extract_/Volume_bioassay_ (Escher et al. 2015). The extract was tested in triplicate at five different REFs (0.01, 0.1, 1, 5, and 10) while a recombined extract (i.e., extract reconstituted from the single fractions after fractionation) was tested in duplicate (REF 0.3, 1.5, 7.5, 15, and 30). For screening purposes, single fractions were tested in one biological replicate at a final REF of 20, an enrichment factor 2-folds higher than the maximum tested concentration where 100% mortality was achieved in the extract for all biological replicates. The pH and the oxygen content were measured at the beginning and at the end of each test. A negative control (ISO water, 0.1% methanol) and positive control (2.1 mg/L, 3,4-dichloroaniline) were included in each experiment. The carrier solvent used for the test was methanol using a maximal concentration of 0.1% (v/v) shown previously to exhibit no effect on the test system (Välitalo et al. 2017). The mortality rate of all negative and positive controls was in the expected range (≤ 10% and ≥ 50% respectively).

### AChE inhibition assay with *Danio rerio* eleutheroembryos

The in vivo AChE assay followed the method described by Küster (2005). Briefly, 10 zebrafish eleutheroembryos (96 hpf) were exposed to the extract and its 26 fractions for 96 h.

After exposure, organisms without lethal and sub-lethal effects were polled and homogenized in 200 µL ice-cold phosphate buffer (pH 7.5, 0.1 M NaH_2_PO_4_·H_2_O-containing 0.1% Triton X-100). Homogenization was performed by keeping samples on ice and using a FastPrep®-24 device (MP Biomedicals) for a maximum of 10 s. Samples were then centrifuged (13,000 × g, 30 min, 4 °C); the supernatant was removed and used either directly for enzyme analysis or stored at − 80 °C until analysis. Storage of the samples never exceeded 6 weeks. The extract was tested at a concentration corresponding to the estimated LC_10_ (REF 2.8) obtained from the FET described in the previous section. To account for potential losses during fractionation, single fractions were tested at a concentration 10 times the estimated LC_10_ (REF 30). All enzyme assays and protein determinations were carried out in different replicates (2 and up to 4 for active fractions, 6 for negative controls) at 22 °C. AChE experiments were performed in four independent exposures, each one including a negative and positive control plus a different number of treatments (extracts and its fractions).

Each AChE test was run with a freshly prepared positive control (eserine hemisulfate, CAS 64–47-1, 50 µM).

### Data handling and statistical analysis

Statistically significant differences between control and treatments for AChE inhibition were estimated using an ANOVA with Dunnett’s post hoc test using the software GraphPad Prism (version 8.4.2). Data obtained from four independent experiments were combined into one dataset before statistical analyses. Data pooling was performed by assuming no relevant difference of AChE activity among negative controls run in each experiment. In the present study, negative controls’ standard deviation of AChE activity did not exceed 22%, a biological variation in line with the ones that were previously observed in zebrafish eleutheroembryos by other authors using a colorimetric determination (Kais et al. 2015; Rodríguez-Fuentes et al. 2015; Sandoval-Gío et al. 2021). Differences were defined to be significant for *p* < 0.05.

Effect values for lethality were estimated using equation for a hill curve with a variable slope using the software Sigma Plot (version 14.5). Minimum effect was constrained to 0 and maximum was constrained to 100%. The LC_50_ and the hill slope were determined to characterize the curve. Data handling was performed using Microsoft Excel 2010®.

## Results

### Acute toxicity in the extract and fractions

**Fig. 2:**
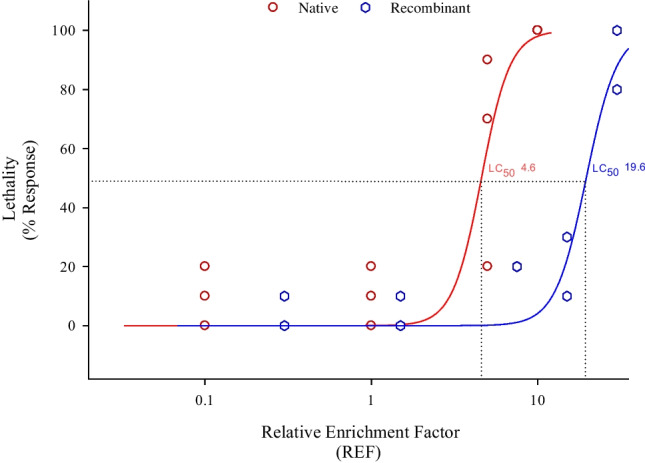
Concentration–response curves for the native (red) and recombinant sample (blue). Lines represent the modelled logistic regression curves. The fish acute toxicity test (FET) was performed in triplicates (native) and duplicate (recombinant). *X*-axis is log-scaled

**Fig. 3:**
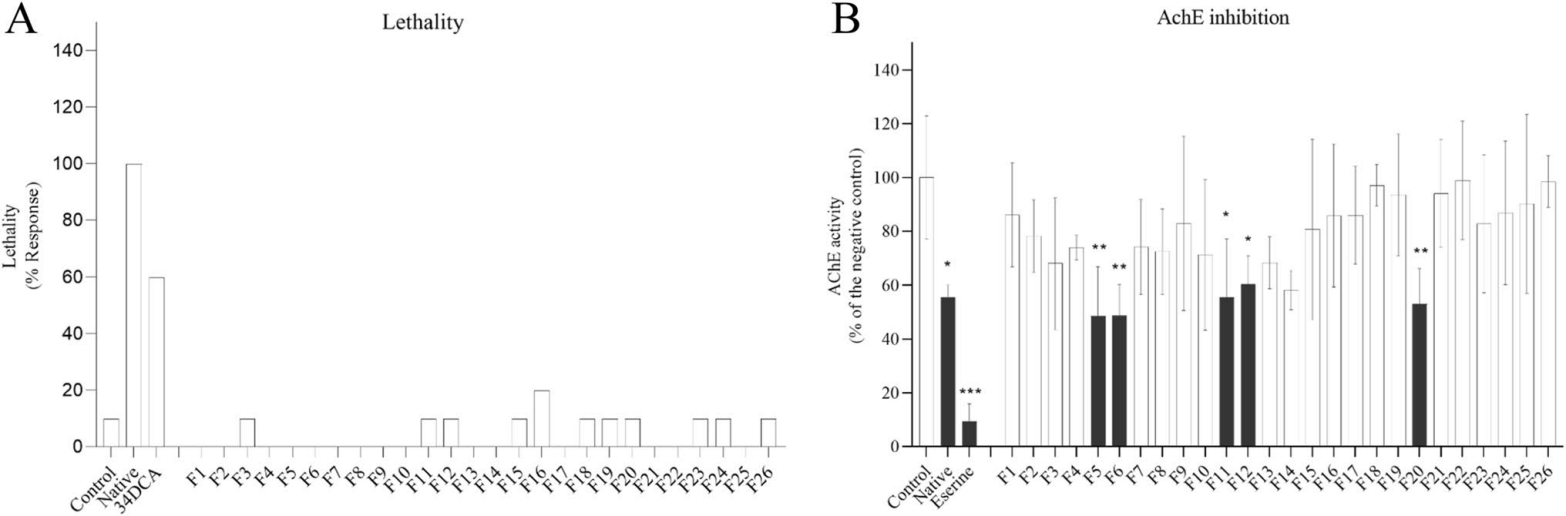
Effects on lethality (**A**) and inhibition of the acetylcholinesterase (AChE) enzyme activity (B) in zebrafish embryos (96 h post fertilization) after exposure to the Novi Sad extract and 26 single fractions (**F**). Fractions for lethality were tested at a REF of 21, while fractions for AChE inhibition were tested at a REF of 30. Black bars represent the fractions statistically different from negative control (ANOVA Dunnett’s post hoc test, *p < 0.05, **p < 0.01, ***p < 0.001). Data are expressed in mean ± SD. Experiments for the AChE assay were performed in different replicates (*n* = 3–4 for active fractions). Negative controls for AChE test were performed in 6 replicates. Positive controls for AChE were performed using the AChE inhibitor eserine hemisulfate (50 µM). Positive controls for mortality were performed using 3,4-dichloroaniline (3,4-DCA, 2.1 mg/L)

The acute FET with zebrafish embryos showed a high sensitivity for the extract with a calculated LC_50_ of REF 4.6 and LC_10_ of REF 2.8 meaning that the extract only had to be slightly concentrated by a factor of 4.6 and 2.8 to have lethal and sub-lethal effects in the FET (Fig. 2). Although the most frequently observed sub-lethal effect was pericardial edema (data not shown), none of the recorded malformations were concentration dependent. Single fractions were tested up to a concentration of REF 20, which is 2 times higher the concentration causing maximum mortality in the extract (REF 10). Mortality or deformations were observed in the larvae in a similar range of negative control (10%) (Fig. 3 A). After recombining the fractions, an LC_50_ of REF 19.6 (Fig. 2) could be calculated. A decrease of the LC_50_ of 4.2 after fractionation was considered in an acceptable range considering the biological variability of in vivo assays as well as possible slight changes of the extract compositions due to different handling steps during extraction, evaporation, and recombination of fractions.

### AChE inhibition in the extract and fractions

The AChE assay showed an inhibition of 45% (*p* = 0.0335) at the calculated LC_10_ for the extract. This result supports the presence of AChE inhibitors in the extract (Fig. 3B). The 26 single fractions were tested up to a concentration of REF 30, which is 10 times higher than the calculated LC_10_. We detected a significant inhibition of the AChE activity in five of the 26 fractions: fractions #5 (52%, *p* < 0.01), #6 (52% inhibition, *p* < 0.01), #11 (45% inhibition, *p* = 0.134), #12 (40% inhibition, *p* = 0.042), and #20 (48% inhibition, *p* < 0.01). Figure 3B shows an overview on the AChE inhibition in the extract and individual fractions.

### Chemical screening and detection of AChE inhibitors in the active fractions

Among the AChE inhibitors detected by König et al. (2017), two were present in active fraction #12. The organophosphate flame-retardant tris(2-chloroethyl) phosphate (TCEP) was detected at a concentration of 1.9 ng/L, while the organophosphate diazinon occurred at concentrations below the method detection limit (MDL) of 1 ng/L. No known AChE inhibitors were detected in the other active fractions. None of the AChE inhibitors in our suspect target list was detected in any of the active fractions.

## Discussion and conclusions

Although the zebrafish FET was originally designed to determine the acute toxicity of chemicals and effluents, modified versions of the FET have been widely used for the toxicological characterization of environmental samples (Hollert et al. 2003; Massei et al. 2019; Serra et al. 2020; Stengel et al. 2018). However, unspecific effects occur after exposure to a very broad range of compounds rather than individual pollutants (Higley et al. 2012; Kammann et al. 2004; Legler et al. 2011). In our study, lethality was observed when embryos were exposed to the extract. However, acute effects tended to disappear when embryos were exposed to single fractions (Fig. 3A) and they appeared again after recombining fractions (Fig. 2). This is not surprising considering that individual fractions represent dilutions of the whole extract. These results agree with previous studies with zebrafish embryos where acute effects tend to disappear after fractionation of environmental samples due to their unspecific nature (Higley et al. 2012; Kammann et al. 2004; Legler et al. 2011).

To successfully detect specific MoAs (e.g., neurotoxicity) in complex environmental mixtures using the FET, it is necessary to use more sensitive and specific biomarkers in addition to the endpoints measured in the FET. Upregulated gene expression and enzymatic assays have been successfully applied for the identification of specific MoAs (Chen et al. 2017; Fang et al. 2014; Fetter et al. 2014; Kammann et al. 2004; Legler et al. 2011; Velki et al. 2017); however, it has been hypothesized that unspecific effects such as oxidative stress may influence non-apical endpoints such as enzymatic activity (de Carvalho Corrêa et al. 2008; den Hartog et al. 2002; Miyagawa et al. 2007; Rico et al. 2007; Schallreuter et al. 2004; Tsakiris et al. 2000; Wyse et al. 2004). Considering this, environmental samples characterized by high chemical loads could also lead to the inhibition of the AChE by inducing oxidative stress. Kais et al. (2015) showed that chemicals with unknown MoAs lead to a different inhibition of the AChE with respect to specific AChE blockers in sediment samples. In line with Kais et al. (2015), our experimental set-up showed that the AChE assay with zebrafish embryos is a specific test able to detect AChE inhibition in complex mixtures as it is confirmed by the recovery of significant effect in five distinct fractions. Our results confirm the analytical potential of the AChE assay with zebrafish for the detection of neuroactive effects in environmental samples.

The FET with zebrafish showed also to have a higher informative power with respect to standalone chemical analysis. The target screening performed by König et al. (2017) confirmed the presence of organophosphate flame retardants (TBOEP and TCPE), organophosphate insecticides (diazinon and ethyl-azinphos), carbamate pesticides (carbaryl and carbendazim), and the food additive caffeine, a non-competitive AChE inhibitor normally detected in high concentrations in raw wastewater (Rousis et al. 2017). Although two compounds (TCPE and diazinon) were detected in an active fraction, concentrations were too low to explain the observed inhibition. Although we cannot exclude that chemical degradation could have occurred during sample storage due to long-term storage, it is reasonable to hypothesize that other AChE inhibitors present in the mixture or their combination were probably driving the effect in the Novi Sad samples. It is well-documented that several pollutants can affect AChE at concentrations below typical chemical analytical detection limits. Highly specific AChE inhibitors such as diazinon and chlorpyrifos were found to influence the AChE in the liver, muscle, and brain of *Cyprinus carpio* and *Chirostoma jordani* at concentrations of 3 ng/L (Dzul-Caamal et al. 2012; Oruc 2011). The carbamate carbofuran inhibits AChE in the brain of *Cyprinus carpio* at 5 ng/L (Dembélé et al. 2000), while chlorpyrifos inhibits AChE in *Hyalella azteca* at a concentration of 0.3 ng/L (Anderson and Lydy 2002). Although we did not find data in literature for such low effect concentrations for zebrafish embryos or larvae, it is important to note that AChE inhibitors may be acting through concentration addition (Mwila et al. 2013). Consequently, several compounds present at sub-ng/L level can work additively and synergistically leading to AChE inhibition.

It may be concluded that standalone target chemical analysis can overlook relevant neuroactive chemicals which are still not included in monitoring lists. In fact, many chemicals enter into the market daily and new compounds are continuously discovered in different environmental matrices (Hashmi et al. 2020). A possible solution would be to implement monitoring with suspect chemical approaches for identification of a wider range of neuroactive chemicals which are commercially available. Recently, a comprehensive list of suspect human neurotoxicants was compiled from different public resources and summarized in a publication from Schymanski et al. (2019). A list just focusing on AChE inhibitors for aquatic species used beyond agriculture (i.e., veterinary and human medicine) may be also compiled in the future for the detection of specific AChE inhibitors in different environmental matrices. To this end, it is also necessary to reach a deeper understanding of toxicological mechanisms underlying the inhibition of the AChE. In fact, several compounds detected by König et al. (2017) could potentially target the AChE without being listed as classic reversible or irreversible inhibitors. In particular, triazine herbicides (e.g., atrazine) showed to potentially inhibit the AChE in zebrafish larvae (Schmidel et al. 2014). Common wastewater pollutants such as surfactants are known to affect the AChE in vivo and in vitro (Guilhermino et al. 2000). Metals are able to inhibit the AChE in zebrafish by binding to thiol groups close to the active site of the enzyme (de Lima et al. 2013). PAHs showed to inhibit the AChE in an in vitro system (Kang and Fang 1997) excluding a contribution from oxidative stress. In fact, it has also been reported that also oxidative stress might play a role in the regulation of the AChE (Rodríguez-Fuentes et al. 2015; Rico et al. 2007). In this case, it would of great support to apply further in vivo measurement of catalase activities and staining to exclude the contribution of oxidative stress to the AChE inhibition and confirm the presence of AChE inhibitors in the environmental mixture. As suggested by Kais et al. (2015), future research should be conducted on additional mechanisms that influence the AChE activity. It was recently demonstrated by Işık (2019) the potential in vivo AChE inhibitory effect of anesthetic agents (i.e., midazolam, propofol, and thiopental). This confirm that studies focusing on predicting binding mechanisms, molecular docking, and kinetic analysis might help to discover new AChE inhibitors and their additional toxicological mechanisms.

## Supplementary Information

Below is the link to the electronic supplementary material.

Supplementary file1 (DOCX 33.9 KB)

## Data Availability

Raw data, associated meta data, and calculation tools are stored according to the guidelines of good scientific practice in the internal archives of the Helmholtz Centre for Environmental Research—UFZ.

## References

[CR1] Altenburger R, Brack W, Burgess RM, Busch W, Escher BI, Focks A, Hewitt LM, Jacobsen BN, de Alda ML, Ait-Aissa S (2019). Future water quality monitoring: improving the balance between exposure and toxicity assessments of real-world pollutant mixtures. Environ Sci Eur.

[CR2] Anderson TD, Lydy MJ (2002). Increased toxicity to invertebrates associated with a mixture of atrazine and organophosphate insecticides. Environ Toxicol Chem: An Int J.

[CR3] Brack W, Aissa SA, Backhaus T, Dulio V, Escher BI, Faust M, Hilscherova K, Hollender J, Hollert H, Müller C (2019). Effect-based methods are key. The European collaborative project solutions recommends integrating effect-based methods for diagnosis and monitoring of water quality. Environ Sci Eur.

[CR4] Casida JE (2009). Pest toxicology: the primary mechanisms of pesticide action. Chem Res Toxicol.

[CR5] Casida JE, Durkin KA (2015). Novel gaba receptor pesticide targets. Pestic Biochem Physiol.

[CR6] Čelić Mira (2020). Occurrence and assessment of environmental risks of endocrine disrupting compounds in drinking, surface and wastewaters in Serbia. Environ Pollut.

[CR7] Chen Q, Gundlach M, Yang S, Jiang J, Velki M, Yin D, Hollert H (2017). Quantitative investigation of the mechanisms of microplastics and nanoplastics toward zebrafish larvae locomotor activity. Sci Total Environ.

[CR8] de Carvalho Corrêa CM, Maldonado P, da Rosa CS, Lunkes G, Lunkes DS, Kaizer RR, Ahmed M, Morsch VM, Pereira ME, Schetinger MR (2008). Oxidative stress and erythrocyte acetylcholinesterase (ache) in hypertensive and ischemic patients of both acute and chronic stages. Biomed Pharmacother.

[CR9] de Lima D, Roque GM, de Almeida EA (2013). In vitro and in vivo inhibition of acetylcholinesterase and carboxylesterase by metals in zebrafish (danio rerio). Mar Environ Res.

[CR10] Dembélé K, Haubruge E, Gaspar C (2000). Concentration effects of selected insecticides on brain acetylcholinesterase in the common carp (cyprinus carpio l.). Ecotoxicol Environ Saf.

[CR11] den Hartog GJ, Vegt E, van der Vijgh WJ, Haenen GR, Bast A (2002). Hypochlorous acid is a potent inhibitor of acetylcholinesterase. Toxicol Appl Pharmacol.

[CR12] Di Paolo NC, Shafiani S, Day T, Papayannopoulou T, Russell DW, Iwakura Y, Sherman D, Urdahl K, Shayakhmetov DM (2015). Interdependence between interleukin-1 and tumor necrosis factor regulates TNF-dependent control of Mycobacterium tuberculosis infection. Immunity.

[CR13] Dutta HM, Arends DA (2003). Effects of endosulfan on brain acetylcholinesterase activity in juvenile bluegill sunfish. Environ Res.

[CR14] Dzul-Caamal R, Domínguez-López ML, García-Latorre E, Vega-López A (2012). Implications of cytochrome 450 isoenzymes, aryl-esterase and oxonase activity in the inhibition of the acetylcholinesterase of chirostoma jordani treated with phosphorothionate pesticides. Ecotoxicol Environ Saf.

[CR15] Escher BI, Neale PA, Leusch FD (2015). Effect-based trigger values for in vitro bioassays: reading across from existing water quality guideline values. Water Res.

[CR16] Fang M, Getzinger GJ, Cooper EM, Clark BW, Garner LV, Di Giulio RT, Ferguson PL, Stapleton HM (2014). Effect-directed analysis of Elizabeth River porewater: developmental toxicity in zebrafish (danio rerio). Environ Toxicol Chem.

[CR17] Fetter E, Krauss M, Brion F, Kah O, Scholz S, Brack W (2014). Effect-directed analysis for estrogenic compounds in a fluvial sediment sample using transgenic cyp19a1b-gfp zebrafish embryos. Aquat Toxicol.

[CR18] Fu H, Xia Y, Chen Y, Xu T, Xu L, Guo Z, Xu H, Xie HQ, Zhao B (2018). Acetylcholinesterase is a potential biomarker for a broad spectrum of organic environmental pollutants. Environ Sci Technol.

[CR19] Guilhermino L, Lacerda M, Nogueira A, Soares A (2000). In vitro and in vivo inhibition of daphnia magna acetylcholinesterase by surfactant agents: possible implications for contamination biomonitoring. Sci Total Environ.

[CR20] Hashmi MAK, Escher BI, Krauss M, Teodorovic I, Brack W (2018). Effect-directed analysis (eda) of Danube River water sample receiving untreated municipal wastewater from Novi Sad, Serbia. Sci Total Environ.

[CR21] Hashmi MAK, Krauss M, Escher BI, Teodorovic I, Brack W (2020). Effect-directed analysis of progestogens and glucocorticoids at trace concentrations in river water. Environ Toxicol Chem.

[CR22] Higley E, Grund S, Jones PD, Schulze T, Seiler TB, Varel UL-v, Brack W, Wölz J, Zielke H, Giesy JP (2012). Endocrine disrupting, mutagenic, and teratogenic effects of upper Danube River sediments using effect-directed analysis. Environ Toxicol Chem.

[CR23] Hollert H, Keiter S, König N, Rudolf M, Ulrich M, Braunbeck T (2003). A new sediment contact assay to assess particle-bound pollutants using zebrafish (danio rerio) embryos. J Soils Sediments.

[CR24] Işık M (2019). The binding mechanisms and inhibitory effect of intravenous anesthetics on AChE in vitro and in vivo: kinetic analysis and molecular docking. Neurochem Res.

[CR25] Kais B, Stengel D, Batel A, Braunbeck T (2015). Acetylcholinesterase in zebrafish embryos as a tool to identify neurotoxic effects in sediments. Environ Sci Pollut Res.

[CR26] Kammann U, Biselli S, Hühnerfuss H, Reineke N, Theobald N, Vobach M, Wosniok W (2004). Genotoxic and teratogenic potential of marine sediment extracts investigated with comet assay and zebrafish test. Environ Pollut.

[CR27] Kang J-J, Fang H-W (1997). Polycyclic aromatic hydrocarbons inhibit the activity of acetylcholinesterase purified from electric eel. Biochem Biophys Res Commun.

[CR28] Kimmel CB, Ballard WW, Kimmel SR, Ullmann B, Schilling TF (1995). Stages of embryonic development of the zebrafish. Dev Dyn.

[CR29] König M, Escher BI, Neale PA, Krauss M, Hilscherová K, Novák J, Teodorović I, Schulze T, Seidensticker S, Hashmi MAK (2017). Impact of untreated wastewater on a major European river evaluated with a combination of in vitro bioassays and chemical analysis. Environ Pollut.

[CR30] Küster E (2005). Cholin- and carboxylesterase activities in developing zebrafish embryos (Danio rerio) and their potential use for insecticide hazard assessment. Aquat Toxicol.

[CR31] Legler J, van Velzen M, Cenijn PH, Houtman CJ, Lamoree MH, Wegener JW (2011). Effect-directed analysis of municipal landfill soil reveals novel developmental toxicants in the zebrafish danio rerio. Environ Sci Technol.

[CR32] Legradi J, Di Paolo C, Kraak M, Van der Geest H, Schymanski E, Williams A, Dingemans M, Massei R, Brack W, Cousin X (2018). An ecotoxicological view on neurotoxicity assessment. Environ Sci Eur.

[CR33] Leuthold D, Klüver N, Altenburger R, Busch W (2018). Can environmentally relevant neuroactive chemicals specifically be detected with the locomotor response test in zebrafish embryos?. Environ Sci Technol.

[CR34] Massei R, Hollert H, Krauss M, von Tümpling W, Weidauer C, Haglund P, Küster E, Gallampois C, Tysklind M, Brack W (2019). Toxicity and neurotoxicity profiling of contaminated sediments from gulf of Bothnia (Sweden): a multi-endpoint assay with zebrafish embryos. Environ Sci Eur.

[CR35] Miyagawa K, Narita M, Narita M, Akama H, Suzuki T (2007). Memory impairment associated with a dysfunction of the hippocampal cholinergic system induced by prenatal and neonatal exposures to bisphenol-a. Neurosci Lett.

[CR36] Mwila K, Burton M, Van Dyk J, Pletschke B (2013). The effect of mixtures of organophosphate and carbamate pesticides on acetylcholinesterase and application of chemometrics to identify pesticides in mixtures. Environ Monit Assess.

[CR37] Nagel R (2002). DarT: the embryo test with the zebrafish Danio rerio—a general model in ecotoxicology and toxicology. Altex.

[CR38] OECD (2013) Technical Guidance Document no. 236: fish embryo acute toxicity (FETet) test

[CR39] Oruc E (2011). Effects of diazinon on antioxidant defense system and lipid peroxidation in the liver of cyprinus carpio (l.). Environ Toxicol.

[CR40] Reineke N, Bester K, Hühnerfuss H, Jastorff B, Weigel S (2002). Bioassay-directed chemical analysis of River Elbe surface water including large volume extractions and high performance fractionation. Chemosphere.

[CR41] Rico EP, Rosemberg DB, Dias RD, Bogo MR, Bonan CD (2007). Ethanol alters acetylcholinesterase activity and gene expression in zebrafish brain. Toxicol Lett.

[CR42] Rodríguez-Fuentes G (2015). Impacts of oxidative stress on acetylcholinesterase transcription, and activity in embryos of zebrafish (Danio rerio) following Chlorpyrifos exposure. Comp Biochem Physiol Part C: Toxicol Pharmacol.

[CR43] Rousis NI, Gracia-Lor E, Zuccato E, Bade R, Baz-Lomba JA, Castrignanò E, Causanilles A, Covaci A, de Voogt P, Hernàndez F, Kasprzyk-Hordern B, Kinyua J, McCall AK, Plósz BG, Ramin P, Ryu Y, Thomas KV, van Nuijs A, Yang Z, Castiglioni S (2017). Wastewater-based epidemiology to assess pan-European pesticide exposure. Water Res.

[CR44] Sandoval-Gío, Juan José, et al (2021) Effect of Benzophenone-3 to Acetylcholinesterase and Antioxidant System in Zebrafish (Danio rerio) Embryos. Bull Environ Contam Toxicol 1–6 10.1007/s00128-021-03277-6 34129062

[CR45] Schallreuter KU, Elwary SM, Gibbons NC, Rokos H, Wood JM (2004). Activation/deactivation of acetylcholinesterase by h2o2: more evidence for oxidative stress in vitiligo. Biochem Biophys Res Commun.

[CR46] Schmidel AJ, Assmann KL, Werlang CC, Bertoncello KT, Francescon F, Rambo CL, Beltrame GM, Calegari D, Batista CB, Blaser RE (2014). Subchronic atrazine exposure changes defensive behaviour profile and disrupts brain acetylcholinesterase activity of zebrafish. Neurotoxicol Teratol.

[CR47] Schreiber R, Altenburger R, Paschke A, Schüürmann G, Küster E (2009). A novel in vitro system for the determination of bioconcentration factors and the internal dose in zebrafish (Danio rerio) eggs. Chemosphere.

[CR48] Schulze T, Ahel M, Ahlheim J, Aït-Aïssa S, Brion F, Di Paolo C, Froment J, Hidasi AO, Hollender J, Hollert H (2017). Assessment of a novel device for onsite integrative large-volume solid phase extraction of water samples to enable a comprehensive chemical and effect-based analysis. Sci Total Environ.

[CR49] Schymanski EL, Baker NC, Williams AJ, Singh RR, Trezzi J-P, Wilmes P, Kolber PL, Kruger R, Paczia N, Linster CL (2019). Connecting environmental exposure and neurodegeneration using cheminformatics and high resolution mass spectrometry: potential and challenges. Environ Sci Process Impacts.

[CR50] Serra H, Brion F, Chardon C, Budzinski H, Schulze T, Brack W, Aït-Aïssa S (2020) Estrogenic activity of surface waters using zebrafish-and human-based in vitro assays: the Danube as a case-study. Environ Toxicol Pharmacol 103401 10.1016/j.etap.2020.103401 32417722

[CR51] Stengel D, Wahby S, Braunbeck T (2018). In search of a comprehensible set of endpoints for the routine monitoring of neurotoxicity in vertebrates: sensory perception and nerve transmission in zebrafish (danio rerio) embryos. Environ Sci Pollut Res.

[CR52] Strähle U, Scholz S, Geisler R, Greiner P, Hollert H, Rastegar S, Schumacher A, Selderslaghs I, Weiss C, Witters H (2012). Zebrafish embryos as an alternative to animal experiments—a commentary on the definition of the onset of protected life stages in animal welfare regulations. Reprod Toxicol.

[CR53] Tsakiris S, Angelogianni P, Schulpis KH, Stavridis JC (2000). Protective effect of l-phenylalanine on rat brain acetylcholinesterase inhibition induced by free radicals. Clin Biochem.

[CR54] Välitalo P, Massei R, Heiskanen I, Behnisch P, Brack W, Tindall AJ, Du Pasquier D, Küster E, Mikola A, Schulze T (2017). Effect-based assessment of toxicity removal during wastewater treatment. Water Res.

[CR55] Velki M, Meyer-Alert H, Seiler T-B, Hollert H (2017). Enzymatic activity and gene expression changes in zebrafish embryos and larvae exposed to pesticides diazinon and diuron. Aquat Toxicol.

[CR56] Wyse AT, Stefanello FM, Chiarani F, Delwing D, Wannmacher CM, Wajner M (2004). Arginine administration decreases cerebral cortex acetylcholinesterase and serum butyrylcholinesterase probably by oxidative stress induction. Neurochem Res.

