# Supplementary material for: Neurotoxicity in complex environmental mixtures—a case-study at River Danube in Novi Sad (Serbia) using zebrafish embryos
Source: Environ Sci Pollut Res Int. 2023 Aug 11;30(42):96138–46. doi: 10.1007/s11356-023-29186-1 (PMC10482774; doi:10.1007/s11356-023-29186-1)
Supplement: Supplementary file 1 — Supplementary file1 (DOCX 33.9 KB) [file 11356_2023_29186_MOESM1_ESM.docx]

**SUPPLEMENT INFORMATIONS**

NEUROTOXICITY IN COMPLEX ENVIRONMENTAL MIXTURES – A CASE-STUDY AT RIVER DANUBE IN NOVI SAD (SERBIA) USING ZEBRAFISH EMBRYOS

Riccardo Massei ^1,4^, Werner Brack ^2, 3^, Sven Seidensticker ^5^, Henner Hollert ^3^, Melis Muz ^2^, Tobias Schulze ^2^, Martin Krauss ^2^, Eberhard Küster^1^

^1^ UFZ - Helmholtz Centre for Environmental Research, Department of Bioanalytical ecotoxicology, Leipzig, Germany

^2^ UFZ - Helmholtz Centre for Environmental Research, Department Effect-Directed Analysis, Leipzig, Germany

^3^ Department Evolutionary Ecology and Environmental Toxicology, Faculty Biological Sciences, Goethe University Frankfurt, Frankfurt, Germany

^4^ UFZ - Helmholtz Centre for Environmental Research, Department of Monitoring and Exploration technologies, Leipzig, Germany

^5^ LUFA Nord-West, Institu für Lebensmittelqualität, Oldenburg, Germany

*Address of corresponding author: riccardo.massei@ufz.de

***ANNEX 1***

*Details on fractionation, sample preparation, chemical analysis, target and suspect screening*

*Fractionation procedure.* Ten mL of the extract were evaporated via a gentle nitrogen stream and re-dissolved in 1 mL of methanol to reach a relative enrichment factor (REF) of 10000. Aliquots of 100 μL were fractionated on an octadecyl silica (C18) column (Nucleodur C18 Gravity, size: 250 × 10 mm, 5 μm particle size, Macherey -Nagel, Düren, Germany). A gradient elution was performed using water and methanol, both containing 0.1 % of formic acid at a flow rate of 2.36 mL/min. The gradient was: 0-4 min, 50 % methanol, 4-45 min, 50-95% methanol with linear increase, 45-65 min 95% methanol, and 65-66 min, 95-50% methanol and column equilibration. Samples and blanks were separated into 26 fractions of two-minute intervals. In order to check for losses during fractionation, aliquots of all fractions were recombined and subjected to the same biotesting procedure as the fractions and the parent sample. The recombined sample was reduced to nearly dryness via rotary evaporation and afterwards resolved in 70 μL of gradient grade methanol (final REF: 30000). Each of the 26 fractions was diluted with the required amount of water to achieve a methanol content of 20 % (v/v). Subsequently the fractions were frozen at -80°C and freeze-dried. After freeze-drying the fractions were dissolved in 8 mL of methanol and stored at -20°C in 10 mL amber vials before chemical and biological analyses.

*Sample preparation for chemical analyses.* Each sample was diluted to a final REF of 500 in a mix of methanol and water (70:30) and 10 μL of an internal standard (1 µg/mL) mixture containing 40 isotope-labeled compounds were added to each sample for matrix effect correction (final IS concentration: 10 ng/mL). LC-HRMS/MS analyses were performed on a quadrupole-Orbitrap MS (QExactive Plus, Thermo) ThermoUltimate 3000 LC system with a Kinetex 2.6 µm EVO C18 (50x2.1 mm) column equipped with a pre-column (C18 EVO 5.x2.1 mm) and an inline filter was used for chromatographic separation. A water-methanol mobile phase gradient elution was used at a flow rate of 300 µL min^-1^. Both eluents contained 0.1% formic acid (v/v). The column oven was heated to 40°C and a volume of 10 µL was injected. Analyses were conducted in separate positive mode runs using electrospray ionization combining a full scan experiment (100-1000 m/z) at a nominal resolving power of 70000 (referenced to m/z 200) and data-independent MS/MS experiments at a nominal resolving power of 35000. For the latter, we acquired the data using broad isolation windows of about 50 (i.e., m/z ranges 97-147, 144-194, 191-241, 238-288, 285-335, 332-382, 379-429, 426-476) and 280 (i.e., m/z ranges 460-740, 730).

*Target screening.* For target screening, eight solvent calibration standards (concentrations: 0.5, 1, 5, 10, 50, 100, 500, and 1000 ng/mL) were measured and concentrations were calculated with a minimum of five calibration points. The software TraceFinder 3.2 (Thermo Fisher) was used for data evaluation of the HRMS spectra.

*Suspect screening.* For suspect screening, [M + H]^+^ adduct peaks of the compounds in the suspect list (Table 2) were searched manually with a mass deviation of 5 ppm using the software XCalibur (Thermo Fisher). A peak intensity of 10^5^ was set as threshold and any peak below this threshold was excluded from the subsequent data processing. Since none of the masses were found in any of active fractions or native sample, we could not proceed further with any identification effort.

TABLEs

Table S1. Suspect list of acetylcholinesterase inhibitors. Log D data were retrieved from PubChem (https://pubchem.ncbi.nlm.nih.gov/).

| Compound | Molecular  Formula | CAS | Class | logD | Exact  Mass | Base Peak  [M+H]^+^ |
| --- | --- | --- | --- | --- | --- | --- |
| Parathion | C_10_H_14_NO_5_PS | 56-38-2 | Organophosphate | 3.8 | 291.0330 | 292.0403 |
| Paraoxon | C_10_H_14_NO_6_P | 311-45-5 |  | 1.9 | 275.1970 | 276.2048 |
| Parathion methyl | C_8_H_10_NO_5_PS | 298-00-0 |  | 2.9 | 263.0017 | 264.0090 |
| Methylparaoxon | C_8_H_10_NO_6_P | 950-35-6 |  | 1.3 | 247.0245 | 248.0323 |
| Chlorpyrifos | C_9_H_11_Cl_3_NO_3_PS | 2921-88-2 |  | 5.3 | 348.9262 | 349.9346 |
| Chlorpyrifos Oxon | C_9_H_11_Cl_3_NO_4_P | 5598-15-2 |  | 5.8 | 332.9491 | 333.9569 |
| Chlorpyrifos methyl | C_7_H_7_Cl_3_NO_3_PS | 5598-13-0 |  | 4.3 | 320.8950 | 321.9023 |
| Fospirate | C_7_H_7_Cl_3_NO_4_P | 5598-52-7 |  | 2.4 | 304.9178 | 305.9256 |
| Dichlorvos | C_4_H_7_Cl_2_O_4_P | 62-73-7 |  | 1.4 | 219.9459 | 220.9532 |
| Phosmet | C_11_H_12_NO_4_PS_2_ | 5104-30-3 |  | 2.8 | 316.9945 | 318.0018 |
| Phosmetoxon | C_11_H_12_NO_5_PS | 3735-33-9 |  | 1.4 | 301.0173 | 302.0251 |
| Tetrachlorvinphos | C_10_H_9_Cl_4_O_4_P | 961-11-5 |  | 3.5 | 363.8993 | 364.9065 |
| Azamethiphos | C_9_H_10_ClN_2_O_5_PS | 35575-96-3 |  | 1 | 323.9736 | 324.9815 |
| Terbufos | C_9_H_21_O_2_PS_3_ | 13071-79-9 |  | 4.5 | 288.0441 | 289.0519 |
| Terbufos oxon | C_9_H_21_O_3_PS_2_ | 56070-14-5 |  | 2.9 | 272.0669 | 273.0747 |
| Aldicarb | C_7_H_14_N_2_O_2_S | 116-06-3 | Carbamate | 1.1 | 190.0776 | 191.0853 |
| Carbofuran | C_12_H_15_NO_3_ | 1563-66-2 |  | 2.3 | 222.1125 | 223.1203 |
| Methiocarb | C_11_H_15_NO_2_S | 2032-65-7 |  | 2.9 | 225.0823 | 226.0901 |
| Fenobucarb | C_12_H_17_NO_2_ | 3766-81-2 |  | 2.8 | 207.1259 | 208.1337 |
| Oxamyl | C_7_H_13_N_3_O_3_S | 23135-22-0 |  | -0.5 | 219.0677 | 220.0755 |
| Methomyl | C_5_H_10_N_2_O_2_S | 16752-77-55 |  | 0.6 | 162.0462 | 163.0540 |
| Fenoxycarb | C_17_H_19_NO_4_ | 72490-01-8 |  | 4.3 | 301.1314 | 302.1392 |
